# A balance of Mad and Myc expression dictates larval cell apoptosis and adult stem cell development during *Xenopus* intestinal metamorphosis

**DOI:** 10.1038/cddis.2017.198

**Published:** 2017-05-11

**Authors:** Morihiro Okada, Thomas C Miller, Luan Wen, Yun-Bo Shi

**Affiliations:** 1 Section on Molecular Morphogenesis, Eunice Kennedy Shriver National Institute of Child Health and Human Development, National Institutes of Health, Bethesda, MD, USA

## Abstract

The Myc/Mad/Max network has long been shown to be an important factor in regulating cell proliferation, death and differentiation in diverse cell types. In general, Myc–Max heterodimers activate target gene expression to promote cell proliferation, although excess of c-Myc can also induce apoptosis. In contrast, Mad competes against Myc to form Mad–Max heterodimers that bind to the same target genes to repress their expression and promote differentiation. The role of the Myc/Mad/Max network during vertebrate development, especially, the so-called postembryonic development, a period around birth in mammals, is unclear. Using thyroid hormone (T3)-dependent *Xenopus* metamorphosis as a model, we show here that Mad1 is induced by T3 in the intestine during metamorphosis when larval epithelial cell death and adult epithelial stem cell development take place. More importantly, we demonstrate that Mad1 is expressed in the larval cells undergoing apoptosis, whereas c-Myc is expressed in the proliferating adult stem cells during intestinal metamorphosis, suggesting that Mad1 may have a role in cell death during development. By using transcription activator-like effector nuclease-mediated gene-editing technology, we have generated Mad1 knockout *Xenopus* animals. This has revealed that Mad1 is not essential for embryogenesis or metamorphosis. On the other hand, consistent with its spatiotemporal expression profile, Mad1 knockout leads to reduced larval epithelial apoptosis but surprisingly also results in increased adult stem cell proliferation. These findings not only reveal a novel role of Mad1 in regulating developmental cell death but also suggest that a balance of Mad and Myc controls cell fate determination during adult organ development.

The Myc/Mad/Max network has a vital role in the normal cell cycle, ensuring proper proliferation and differentiation.^1, 2, 3^ c-Myc is a well-characterized transcription factor and oncogene, which generally activates gene transcription, thereby inducing proliferation upon heterodimerizing with Max. Conversely, upon heterodimerizing with Max, Mad strongly represses the transcription of c-Myc target genes, causing cells to exit cell cycle, and is commonly expressed in quiescent or differentiating cells. Whereas c-Myc overexpression can induce cell death,^3, 4, 5, 6, 7, 8, 9, 10, 11, 12^ in many cases Mad has been found to be anti-apoptotic.^1, 3, 13, 14^ Owing to their opposing functions, c-Myc and Mad are not expressed in the same cell and are most often expressed temporally and spatially successively or distinct within tissues.^13, 15, 16^

The metamorphosis of anuran tadpole intestine allows a unique opportunity to examine the importance of Myc/Mad/Max network in controlling cell cycle and cell fate during vertebrate development. Amphibian metamorphosis is totally controlled by thyroid hormone (T3) and resembles postembryonic development in mammals, a period around birth when many organs matured into the adult form.^17^ In *Xenopus laevis* (*X. laevis*) and the highly related species *Xenopustropicalis*, the tadpole intestine is a simple tubular structure made of mainly larval epithelial cells surrounded by thin layers of connective tissue and muscles.^18, 19, 20^ The epithelial cells are fully differentiated into specialized cells yet capable of, and often undergo, mitotic division.^20, 21^ As circulating T3 levels increase, thereby initiating metamorphosis, a subset of these differentiated epithelial cells dedifferentiate into adult stem cells.^22^ The remaining larval epithelial cells undergo apoptosis as new adult tissue expands to completely replace the larval epithelial cells. Importantly, this process resembles the maturation of the adult mammalian intestine around birth when plasma T3 levels are also high,^23, 24^ making it an excellent model to study the formation of organ-specific adult stem cells during postembryonic development.

Previous research has demonstrated that c-Myc is expressed during intestinal metamorphosis and is a direct response gene of T3.^25^ Within the adult mouse intestine, c-Myc is found to be expressed in dividing stem cells and Mad levels increase as cells differentiate and migrate up the crypt–villus axis.^26^ Although Mad expression was localized in terminally differentiated cells during frog embryogenesis,^27^ it remains uncertain if Mad has a similar role in differentiating the expanding adult epithelial stem cells into specialized cells as intestinal metamorphosis is completed in the frog.

Here, we have investigated the function of Mad1 gene during amphibian metamorphosis. We demonstrated that Mad1 expression increased and peaked before c-Myc during T3-induced intestinal metamorphosis. Spatially, Mad1 was located in larval epithelial cells that were not proliferating, whereas c-Myc was present in the proliferating adult epithelial stem cells. By using transcription activator-like effector nuclease (TALEN)-mediating gene-editing *in vivo*, we showed that Mad1 knockout increased levels of proliferating cells and reduced apoptosis during metamorphosis. These findings suggest that Mad1 causes mitotically active larval epithelial cells to exit the cell cycle thereby ensuring their destruction and a Mad–Myc balance controls the expansion of adult intestinal epithelial cells.

## Results

### Mad1 is highly conserved among vertebrate

**Figure 1:**
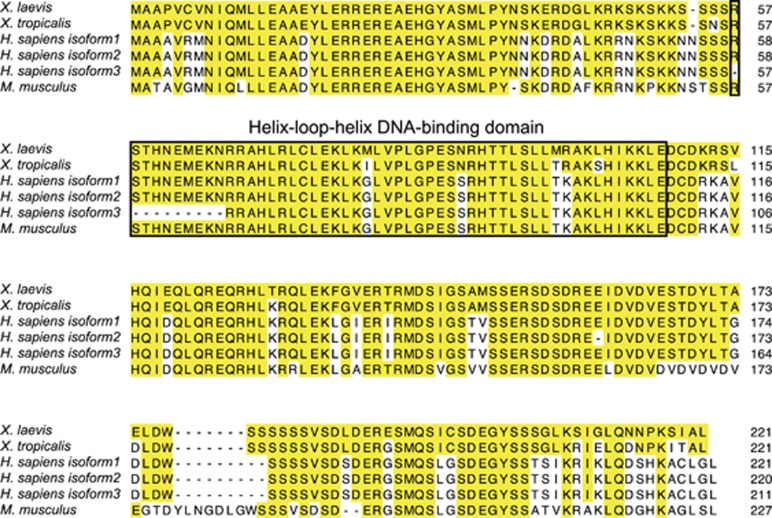
Mad1 is highly conserved in vertebrates. Amino-acid sequence alignment of *X. laevis* (NP_001090200), *X. tropicalis* (NP_001072228), *H. sapiens* (NP_002348, NP_001189442, NP_001189443) and *M. musculus* (NP_034881) Mad1. The shared amino acids are indicated with yellow boxes. The highly conserved HLH DNA-binding domain of Mad1 is boxed

We reported earlier a genome-wide microarray analysis that revealed many *X. laevis* transcripts, including an expressed sequence tag (EST) of an unknown identity, strongly upregulated in the intestinal epithelium during adult stem cell formation.^28^ To identify the EST, we performed rapid amplification of cDNA ends (RACE) analysis, which showed that the EST was a 3700-base pair region in the 3′-UTR of Max dimerization protein 1 (Mxd1) gene, which encodes Mad1 protein (hereto referred to as Mad1). The Mad1 protein is a helix-loop-helix (HLH) transcription factor that competes with c-Myc to heterodimerize with Max. The full-length coding region of *X. laevis* and *X. tropicalis* Mad1 was obtained from GenBank, and amino-acid sequences were thus deduced. Comparison of Mad1 amino-acid sequences from *X. laevis*, *X. tropicalis*, *Homo sapiens* (*H. sapiens*), and *Mus musculus* (*M. musculus*) revealed that Mad1 is highly conserved during evolution with the *X. laevis* Mad1 sequence sharing 94%, 75–79% and 69% homology with that in *X. tropicalis*, *H. sapiens* and *M. musculus*, respectively. Even higher homology was observed within the HLH domain (Figure 1).

### High levels of Mad1 is expressed in the epithelium at the climax of metamorphosis in *X. laevis*

**Figure 2:**
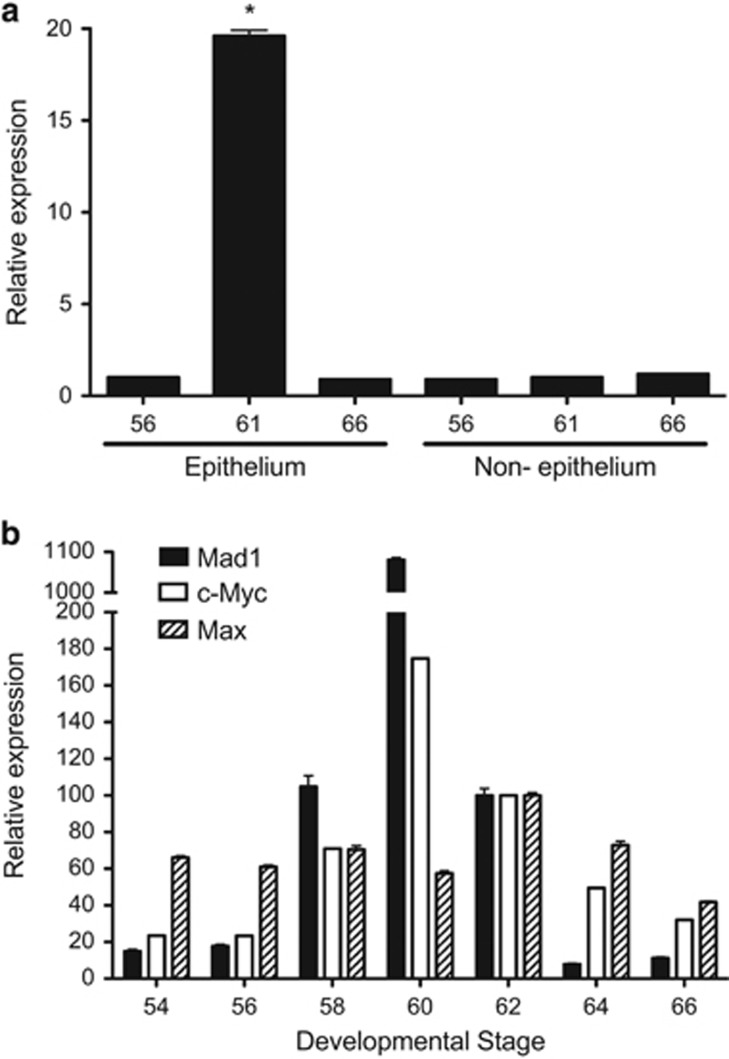
Mad1 is strongly upregulated specifically in the epithelium of the intestine at the climax of metamorphosis when epithelial apoptosis and adult stem cell development take place. (**a**) The expression of Mad1 was analyzed by RT-PCR on total RNA from the isolated epithelium and non-epithelium (the rest of the intestine) of *X. laevis* tadpoles at indicated stages during development. (**b**) Both Mad1 and c-Myc are upregulated, whereas Max expression changes little during intestinal remodeling. The expression of Mad1, c-Myc, and Max was analyzed by RT-PCR on total RNA isolated from whole intestine of *X. laevis* tadpoles at indicated stages during development. The mRNA level for Mad1, c-Myc and Max was normalized against that of EF1*α* RNA. The data are shown in arbitrary unit as the mean±S.E. (*n*=3). The statistical significance was determined by one-way ANOVA followed by Tukey’s multiple comparison test (*P<0.05)

To investigate the tissue specificity of Mad1, we analyzed Mad1 expression by using total RNA isolated from separated epithelium and non-epithelium of the intestine at premetamorphic stage 56, climax stage 61, and end of metamorphosis (stage 66). Consistent with the microarray findings,^28^ Mad1 mRNA had little expression in the epithelium before or after metamorphosis and high levels of expression at the climax of metamorphosis (Figure 2a). Little Mad1 expression was observed in the non-epithelium at any stages (Figure 2a). These results suggest that Mad1 levels temporally correlate with the intestinal epithelial transformation with the vast majority of the larval cells undergoing apoptosis accompanied by the dedifferentiation of some larval epithelial cells into adult stem cells.^19, 29, 30^

Next, we compared the expression profiles of Mad1 and c-Myc during intestinal metamorphosis, RT-PCR was performed with whole intestinal RNA. Mad1 expression was low at premetamorphic stages 54–56 but drastically upregulated during metamorphosis, and reached peak levels at stage 60, when most of the larval epithelial cells are undergoing apoptosis.^19^ The expression subsequently dropped to much lower levels by the end of metamorphosis (stage 66), as the adult epithelial cells differentiate to establish the trough–crest axis of adult epithelial fold (Figure 2b). Interestingly, the expression of c-Myc, which competes against Mad1 to heterodimerize with Max and is known to be regulated by T3 during metamorphosis,^25^ was similarly regulated during intestinal metamorphosis (Figure 2b). On the other hand, the expression of Max changed little during intestinal metamorphosis (Figure 2b). These results suggest that Mad1 and c-Myc are both involved in intestinal remodeling.

### Mad1 expression in the intestine is induced by T3, just before high levels of c-Myc expression

**Figure 3:**
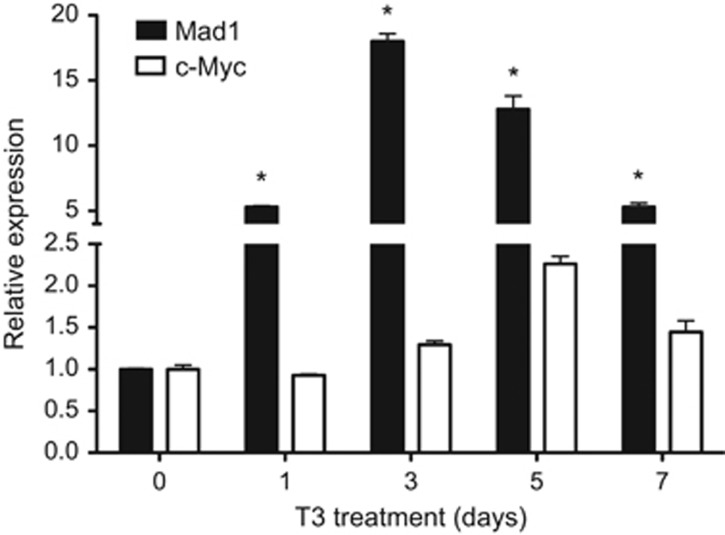
T3 induces Mad1 expression in the intestine, before high levels of c-Myc expression. RT-PCR analysis of Mad1 and c-Myc expression was performed by using total RNA isolated from the intestine of *X. laevis* stage 54 tadpoles treated with 10 nM T3 for indicated numbers of days. The mRNA level for Mad1 and c-Myc gene was normalized against that of EF1*α* mRNA. The data are shown as the mean±S.E. (*n*=3). The statistical significance was determined by one-way ANOVA followed by Tukey’s multiple comparison test (*P<0.05)

Like all other processes during amphibian metamorphosis, intestinal remodeling is under the control of T3. The expression profile of Mad1 during natural metamorphosis suggests that Mad1 is regulated by T3. To investigate this possibility, we analyzed Mad1 expression in the intestine of premetamorphic tadpoles at stage 54 treated with 10 nM T3 for 1–7 days. Mad1 mRNA was rapidly induced by T3 and peaked after 3 days of T3 treatment when larval intestinal epithelial cell death peaks^19, 21^ (Figure 3). On the other hand, c-Myc expression, which is antagonist of Mad1, was moderately induced by T3 and peaked at 5 days of T3 treatment (Figure 3). These findings indicate that Mad expression in the intestine is rapidly induced by T3 and T3-induced Mad1 mRNA is expressed earlier than the massive proliferation of the adult stem cells, which may have high levels of c-Myc expression.

### Mad1 and c-Myc are expressed in distinct epithelial cells during intestinal metamorphosis

**Figure 4:**
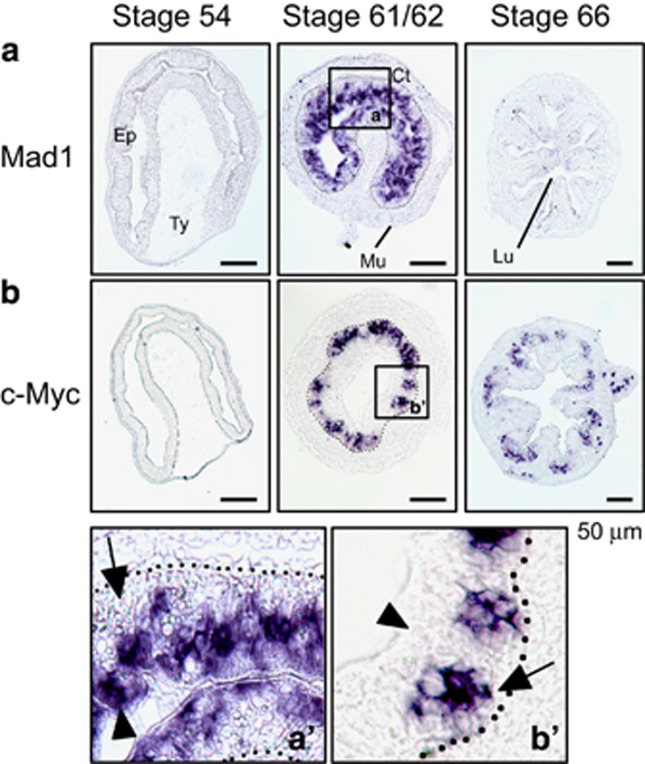
*In situ* hybridization analysis indicates that Mad1 is expressed in the epithelial cells at the climax of intestinal remodeling. Cross-sections of *X. laevis* intestine at premetamorphic stage 54, metamorphic climax stages 61/62 and the end of metamorphosis (stage 66) were hybridized with Mad1 (**a**) and c-Myc (**b**) antisense probe. The boxed region in **a** and **b** at stage 61/62 were enlarged and shown in (**a**′ and **b**′), respectively, at the bottom. Note that Mad1 expression was limited to dying larval epithelial cells facing the intestinal lumen in the epithelium at the climax of metamorphosis, consistent with the expression data in Figure 2. The expression of c-Myc was also high at the climax of metamorphosis but was in the epithelial layer close to the connective tissue. Arrows point to clusters of cells or islets in the epithelium close to the connective interface and expressing c-Myc, whereas arrowheads point to the epithelial cells facing the lumen, expressing Mad1. The approximate epithelium–mesenchyme boundary was drawn based on morphological differences between epithelial cells and mesenchyme cells in the photographs, under enhanced contrast and/or brightness by using Photoshop, if needed (dotted lines). Scale bar, 50 *μ*m. CT, connective tissue; Ep, epithelium; Mu, muscle; Lu, lumen; Ty, typhlosole

To identify Mad1-expressing cells, *in situ* hybridization was carried out on intestinal sections prepared from tadpoles at stages 54 (premetamorphosis), 61/62 (climax) and 66 (end of metamorphosis). Consistent with the RT-PCR analysis shown in Figure 2, little or no Mad1 mRNA was detectable at premetamorphic stage 54 or the end of metamorphosis (stage 66) (Figure 4a). In contrast, high levels of Mad1 mRNA were localized only in the epithelium at the climax of metamorphosis (stage 61/62) (Figure 4a, a′). The c-Myc mRNA was similarly found to be in the epithelium at the climax of metamorphosis (Figure 4b, b′) and lower levels were also found at the base of the epithelial fold at the end of metamorphosis, again consistent with the RT-PCR analysis.

**Figure 5:**
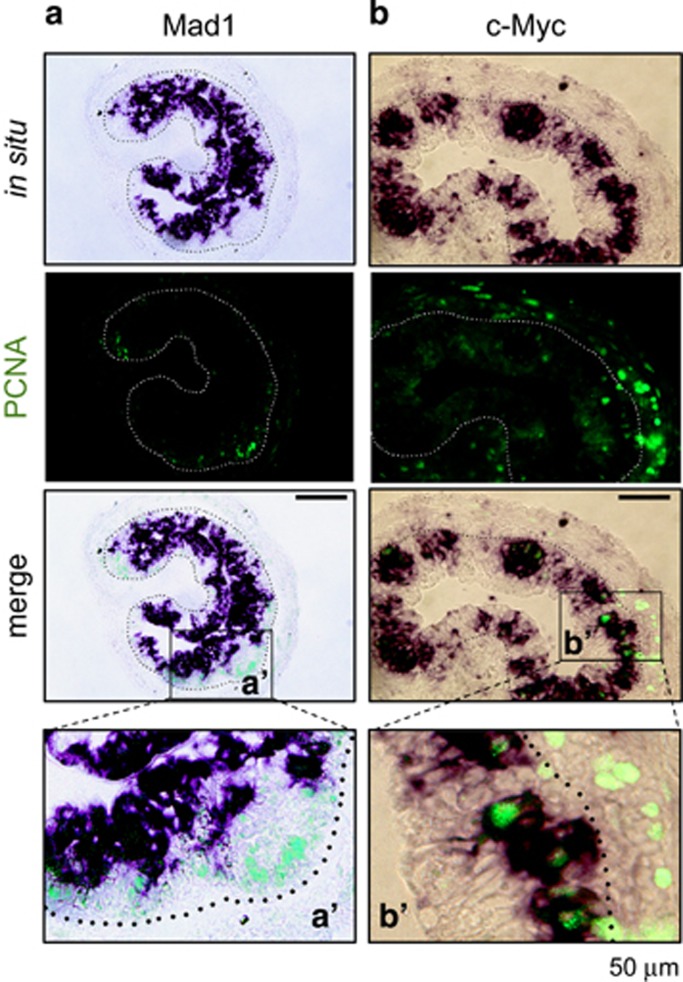
High levels of Mad1 mRNA are expressed in apoptotic but not proliferating epithelial cells. Double staining of PCNA immunohistochemistry (green: cell proliferation) and Mad1 (**a**) or c-Myc (**b**) *in situ* hybridization (purple) was performed on cross-sections of *X. laevis* intestine at metamorphic climax stage 61/62. Note that Mad1 expression was limited to the epithelial cells facing the lumen, which are known to be dying larval epithelial cells at the climax of metamorphosis.^21^ These cells did not have PCNA. The PCNA positive, proliferating cells lacked Mad1 mRNA and were localized in between the dying epithelial cells and the connective tissue. In contrast, c-Myc-expressing cells were found to colocalize with the proliferating cells expressing PCNA (**b**). Higher magnification of boxed areas in the merged panel in (**a** and **b**) are shown in a′ and b′, respectively. The approximate epithelium–mesenchyme boundary was drawn based on morphological differences between epithelial cells and mesenchyme cells in the photographs, under enhanced contrast and/or brightness by using Photoshop, if needed (dotted lines). Note that for unknown reason, we observed that the PCNA signals in the connective tissue in (**b**) were much stronger than in (**a**). It is possible that it was in part because of weaker staining in (**a**) and in part that the section in (**b**) happened to have more proliferating cells. Nonetheless, the colocalization of PCNA with c-Myc but not Mad1 was consistently observed in different intestinal sections. Scale bar, 50 *μ*m

We have previously shown that Lgr5 (a well-known adult stem cell marker of mammalian intestine)-positive cell clusters are numerous in the epithelium at the climax and these clusters are labeled with cell proliferation markers.^21^ To determine if Mad1 mRNA is expressed in the proliferating adult stem cells, we used double labeling to simultaneously detect Mad1 mRNA by *in situ* hybridization and proliferating cell nuclear antigen (PCNA), a marker of cell proliferation, by immunohistochemistry. No co-staining of any cells by Mad1 mRNA and PCNA was detected in the intestine at the climax of metamorphosis (Figure 5a, a′). When we carried out similar double labeling of c-Myc mRNA and PCNA, we observed that c-Myc-positive cell clusters were numerous in the epithelium and these clusters were also labeled with PCNA labeling (Figure 5b, b′). Taken together, these findings indicate that c-Myc is expressed in proliferating adult intestinal cells close to the connective tissue, whereas high levels of Mad1 mRNA are present in the degenerating (non-proliferating) larval epithelial cells facing the lumen.

### Mad1 is not essential for embryogenesis and metamorphosis

**Figure 6:**
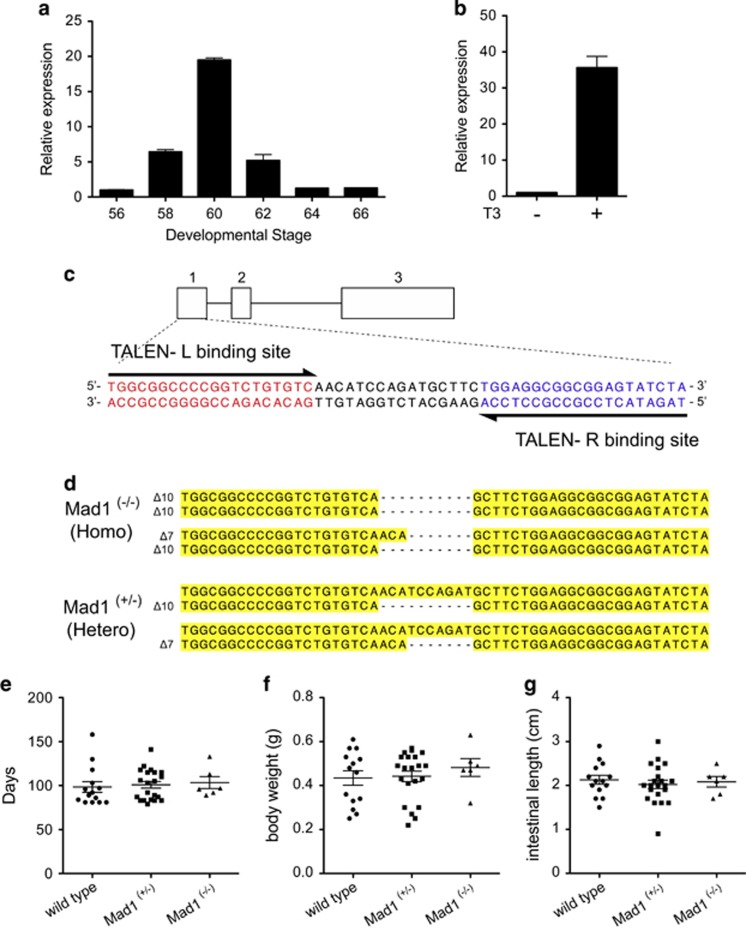
Mad1 knockout tadpoles have no growth defects. (**a**) *X. tropicalis* Mad1 is similarly upregulated during intestinal metamorphosis as in *X. laevis*. The expression of Mad1 was determined by RT-PCR analysis of total RNA isolated from whole intestine of *X. tropicalis* tadpoles at indicated stages during development. Mad1 mRNA level was normalized against that of EF1*α* mRNA. The statistical significance was determined by one-way ANOVA followed by Tukey’s multiple comparison test (*P<0.05). (**b**) T3 induces Mad1 expression in *X. tropicalis* premetamorphic tadpole intestine. Stage 54 *X. tropicalis* tadpoles were treated with or without 10 nM T3 for 2 days. Total RNA was isolated from the intestine and analyzed by RT-PCR for Mad1 expression. Mad1 mRNA level was normalized against that of EF1*α* mRNA. The data are shown as the mean±S.E. (*n*=3). The statistical significance of differences was determined by Student’s *t*-test (*P<0.05). (**c**) Mad1 genomic structure and target sequences of the TALEN against *X. tropicalis* Mad1 locus. There are three exons in *X. tropicalis* Mad1 and Mad1-specific TALEN left (L) and right (R) arms were generated to target exon 1. The left and right TALEN-binding sequences are shown in red and blue, respectively, and the spacer region is in black. (**d**) Sequence of homozygous (Δ10/Δ10, Δ10/Δ7) and heterozygous (+/Δ10, +/Δ7) tadpoles found in F2 generation tadpoles. Deletions are indicated by dashes. (**e**-**g**) No gross defect because of Mad1 knockout is found in animals at the end of metamorphosis. Tadpoles were reared identically to stage 66 and then genotyped. The time to reach stage 66 (**e**), the body weight (**f**) and intestinal length (**g**) at stage 66 for each animal was determined and plotted with the mean, marked as a line, and standard error. Note that no significant difference was observed for any of the parameters

Our spatiotemporal expression data suggest that Mad1 is involved in intestinal metamorphosis, likely in the larval epithelial cell death. To investigate the role of Mad1 during metamorphosis, we adapted the recently developed TALEN technology to knockout the endogenous Mad1. We turned to the *X. tropicalis* model as its genome has been sequenced and it has a diploid genome, contrasting the pseudo-tetraploid nature of the *X. laevis* genome. Furthermore, the species are highly conserved with all genes analyzed so far having similar expression and regulation patterns during metamorphosis in the two species.^18, 31, 32, 33, 34, 35, 36, 37, 38^ Indeed, when we analyzed the expression of Mad1 during intestinal metamorphosis in *X. tropicalis*, we observed that it was also highly upregulated at the climax of metamorphosis (Figure 6a). Similarly, treating premetamorphic *X. tropicalis* tadpoles with T3 led to strong induction of Mad1 in the intestine (Figure 6b).

To knockout Mad1, we designed a TALEN nuclease made of a pair of left and right arms targeting the second ATG codon in exon 1 of *X. tropicalis* Mad1 (Figure 6c). *In vitro* synthesized mRNA encoding the arms were micro-injected into fertilized eggs. Three days after TALEN mRNA injection when the eggs developed into feeding tadpoles (stage 45), we analyzed the mutation rate at the Mad1 locus by using pooled genomic DNA derived from the Mad1 TALEN-injected F0 tadpoles. Mad1 TALEN was found to specifically mutate the endogenous Mad1 gene with 70% efficiency (data not shown). The rest of F0 tadpoles were reared and found to undergo normal metamorphosis and develop into sexually mature adult frogs. Genome typing identified mosaic F0 frogs with high percentages of 7- or 10-nucleotide deletions at the Mad1 targeted region (Figure 6d). The F0 frogs were mated with wild-type frogs to obtain F1 heterozygous animals. F0 males (containing 7- or 10-nucleotide deletion) or F1 males (containing heterozygous 10-nucleotide deletion) and F1 females (containing heterozygous 10-nucleotide deletion) were mated to obtain F2 offspring (Figure 6d). The 7- or 10-nucleotide deletion mutations at the TALEN target site caused frame-shifts and would abolish the HLH domain by creating stop codons. Thus, animals contained homozygous 7- or 10-nucleotide deletion or with a 7-nucleotide deletion in one allele and a 10-nucleotide deletion in the second allele would lack any functional Mad1, in effect creating a total Mad1 knockout (all were referred to, therefore, as homozygous knockout animals). When we analyzed the Mad1 homozygous knockout, heterozygous (with one wild-type copy of Mad1) and wild-type animals, we observed that all animals could development from fertilized eggs (stage 1) to the end of metamorphosis (stage 66). There were no detectable external morphological differences among the three genotypes, including the number of days to reach the end of metamorphosis as judged based on external morphology (Figure 6e), the body weight (Figure 6f), and length of the animal intestine at the end of metamorphosis (Figure 6g). Thus, Mad1 is not essential for embryogenesis and metamorphosis.

### Mad1 knockout reduces larval epithelial apoptosis and enhances adult intestinal epithelial stem cell proliferation during T3-induced metamorphosis

**Figure 7:**
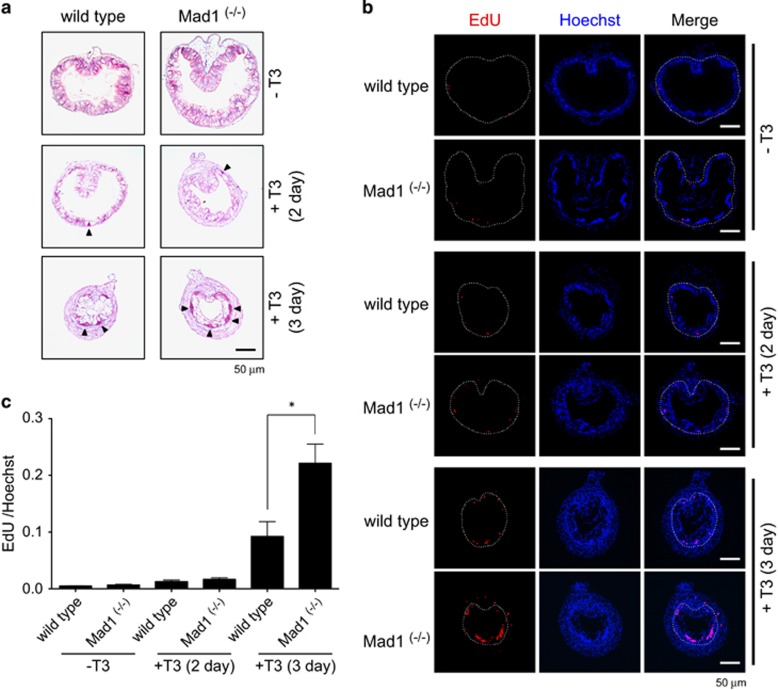
Mad1 knockout enhances intestinal epithelial cell proliferation during T3-induced metamorphosis. Premetamorphic stage 54 tadpoles were treated with 5 nM T3 for 0, 2 or 3 days and were killed 30 min after EdU injection. (**a**) Cross-sections of the intestine from the resulting tadpoles were stained for MGPY. Note there were more epithelial cells in clusters strongly stained by MGPY in the cross-sections of the intestine from Mad1 ^(−/−)^ tadpoles treated with T3 for 3 day compared with wild-type ones. Arrowheads indicate the clusters of epithelial cells. (**b**) Cross-sections of the intestine from the tadpoles were double-stained for EdU (cell proliferation) and with Hoechst (DNA). Again, there are more EdU-positive epithelial cells in clusters in the T3-treated Mad1 ^(−/−)^ tadpole intestinal cross-sections compared with the wild-type ones. The dotted lines depict the epithelium–mesenchyme boundary. Scale bar, 50 *μ*m. (**c**) Cell proliferation is significantly increased in Mad1 ^(−/−)^ tadpoles treated with T3 for 3 day compared with wild-type ones. Red colored EdU positive areas in epithelium were measured and normalized against the total cellular area in epithelium determined by Hoechst staining. The statistical significance of the differences was determined by Student’s *t*-test (*P<0.05)

Given the high levels of Mad1 expression at the climax when larval epithelial apoptosis and adult stem cell development take place,^19^ we next investigated the effects of Mad1 knockout on intestinal stem cell development. For this purpose, we treated premetamorphic tadpoles at stage 54 with T3 for 0–3 days to induce metamorphosis. It is well known that proliferating adult epithelial cells can be easily identified as clusters of cells or islets that can be strongly stained with methyl green-pyronin Y (MGPY) and labeled with 5-ethynyl-2’-deoxyuridine (EdU), a cell proliferation marker.^21^ Thus, we analyzed intestinal cross-sections by using MGPY staining and EdU labeling. The epithelium was uniformly stained with MGPY in premetamorphic intestine after 0 or 2 days of T3 treatment (Figure 7a). After 3 days of T3 treatment, MGPY strongly stained clusters of cells appeared in the epithelium and there were more such cells in Mad1 knockout tadpoles compared with wild-type tadpoles (Figure 7a). Furthermore, EdU labeling showed that there were more proliferating cell clusters in the epithelium of Mad1 knockout tadpoles compared with wild-type tadpoles after treatment with T3 for 3 days (Figures 7b and c), indicating that Mad1 knockout enhances the formation and/or the proliferation of T3-induced adult stem cells.

**Figure 8:**
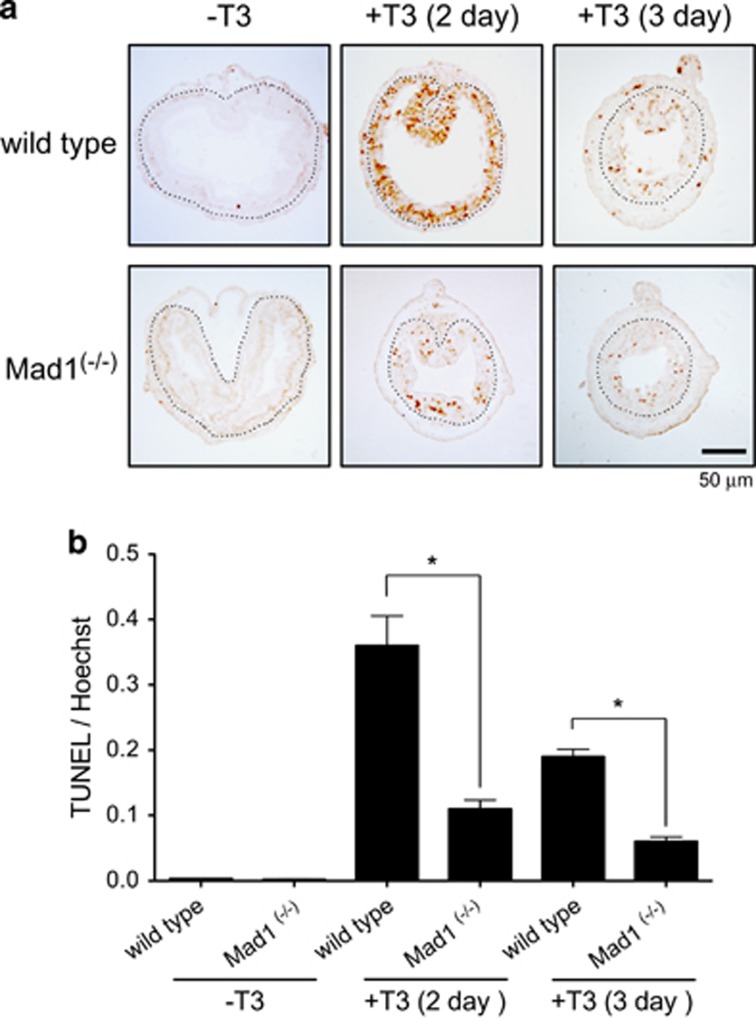
Mad1 knockout inhibits cell death in the epithelium during T3-induced metamorphosis. (**a**) Cross-sections of the intestine of premetamorphic stage 54 tadpoles treated with 5 nM T3 for 0, 2 or 3 days were stained for apoptosis by TUNEL. Note that apoptosis in the epithelium peaked after 2 days of T3 treatment and that there were more intestinal epithelial cells in wild-type animals strongly stained by TUNEL compared with the ones in Mad1 ^(−/−)^ tadpoles. The dotted lines depict the epithelium–mesenchyme boundary. Scale bar, 50 *μ*m. (**b**) Quantitative analysis of apoptosis by counting TUNEL-positive areas in the epithelium and normalized by the total cellular area in epithelium determined by Hoechst staining

We next investigated if Mad1 knockout affected larval epithelial degeneration during T3-induced metamorphosis. Terminal deoxyribonucleotidyl transferase-mediated dUTP-biotin nick end labeling (TUNEL) assay was performed to detect apoptotic cells in the intestine. As shown in Figure 8a, little apoptotic signals were present in the epithelium of premetamorphic intestine. After 2 days of T3 treatment, high levels of apoptotic signals were present in the epithelium of wild-type intestinal epithelium (Figure 8a). In the Mad1 knockout tadpoles, however, the TUNEL signals were much less (Figure 8a). After 3 days of T3 treatment when EdU labeling was the strongest (Figures 7b and c), epithelial cell death began to decrease in the intestine of both wild-type and knockout animals (Figure 8a). Quantification of the TUNEL labeling revealed that knocking out Mad1 led to a threefold reduction in the larval epithelial cell death after treatment with T3 for 2 and 3 days (Figure 8b). Thus, Mad1 is important for both larval cell death and adult epithelial stem cell development during intestinal metamorphosis.

## Discussion

Intestinal remodeling during *Xenopus* metamorphosis offers a unique opportunity to study the formation of adult organ-specific stem cells during postembryonic development in vertebrates in part because of the difficulty to manipulate late stage, uterus-enclosed mammalian embryos.^24^ Although the functions and properties have been studied extensively for different adult organ-specific stem cells, much less is known about how such stem cells are formed during development. Earlier studies in *X. laevis* have shown that adult *Xenopus* intestinal stem cells are formed *de novo* in a T3-dependent process and increasing evidence suggests that the mammalian adult intestinal stem cells are formed similarly around the neonatal period.^24^ Using the *Xenopus* model system, we have previously identified many T3 response genes likely involved in the development of the adult stem cells. Our studies here have revealed a novel function for one of such candidate stem cell genes, Mad1, in regulating larval epithelial cell death to affect adult stem cell development.

Intestinal remodeling during *Xenopus* metamorphosis involves near-complete degeneration of the larval epithelium through programmed cell death and *de novo* development of adult epithelial stem cells.^19, 29, 30^ Importantly, this process is regulated by T3 in an organ-autonomous manner as organ cultures of premetamorphic intestine can be induced to metamorphose with physiological levels of T3.^39, 40^ By using recombinant organ cultures with wild-type and GFP-expressing transgenic tadpole intestine, we have previously demonstrated that the adult intestinal stem cells are derived from larval epithelium through T3-induced dedifferentiation of some larval cells.^41^ In addition, it has been shown that non-epithelial tissues within the intestine, mainly the connective tissue, are important for epithelial cell fate in the presence of T3 and required for adult epithelium development.^39, 40^ More importantly, recombinant organ culture studies with transgenic intestine expressing a constitutively active form of TR have demonstrated that T3 action in the epithelium alone can induce larval cell dedifferentiation but the formation of adult stem cells also require T3 action in the rest of the intestine, most likely the connective tissue underlying the epithelium.^42^ These studies together indicate that the expression of T3 response genes in both the epithelium and non-epithelial tissues are required for stem cell development during metamorphosis. In particular, T3 response genes specifically expressed in the epithelium are likely critical for cell fate determination in the larval epithelium, that is, apoptosis *versus* dedifferentiation in the presence of T3.

As we have shown here, Mad1 is a T3-induced gene specifically expressed in the epithelium during intestinal metamorphosis. Its expression is highly upregulated in the epithelial cells at the climax of metamorphosis when larval epithelial cells undergo apoptosis and adult epithelial stem cells are forming and rapidly proliferating.^19^ Spatially, the dying larval epithelial cells face the intestinal lumen, whereas the proliferating adult stem cells sit in between the dying larval epithelial cells (if still remaining in the epithelium) and the connective tissue. Interestingly, Mad1 mRNA is specifically present in the cells facing the lumen, whereas c-Myc mRNA is present in clusters of cells between the connective tissue and dying epithelial cells. Thus, Mad1-expressing cells are the apoptotic larval cells, whereas c-Myc-expressing cells are the proliferating adult stem cells. Consistently, c-Myc but not Mad1 is co-expressed with PCNA, a cell proliferation marker. Although the lack of appropriate antibodies made it impossible to determine the corresponding protein levels, it is likely that they are similarly regulated spatiotemporally. Thus, our findings suggest that Mad1 may be induced by T3 to promote larval epithelial cell death.

Both c-Myc and Mad1 are members of the Myc/Mad/Max network, which is of critical importance in regulating cell physiology.^1, 2, 3, 13, 14^ Mad1 is one of several Mad proteins that can heterodimerizes with Max, thus, competing against members of the Myc family to regulate target gene expression. It has been well-established that Myc–Max heterodimers are DNA-binding transcription factors that activate target gene expression to promote cell proliferation and that excess of c-Myc can cause programmed cell death or apoptosis.^3, 4, 5, 6, 7, 8, 9, 10, 11, 12^ In contrast, Mad–Max heterodimers bind to the same target genes but repress their expression, thereby functioning as a c-Myc antagonist.^13^ Thus, it is not surprising that in many cases Mad has been found to be anti-apoptotic^1, 3, 13, 14^ and that c-Myc and Mad are not expressed in the same cell.^13, 15, 16^ Consistently, c-Myc is expressed in proliferating adult epithelial stem cells during intestinal metamorphosis. Furthermore, it is upregulated by T3 during metamorphosis. Although the kinetics of c-Myc upregulation by T3 appears to be slow when analyzed at whole intestine level, this may be a reflection of the lack of adult stem cells before T3 treatment. T3 induces *de novo* formation of adult stem cells, which subsequently increase in number, leading to increased overall c-Myc mRNA level in the intestine after prolonged T3 treatment. In fact, our earlier studies have revealed the presence of a T3 response element (TRE) in the regulatory region of the c-Myc promoter and that this TRE is bound by TR in the intestine during metamorphosis. Thus, T3 appears to directly induce the transcription of c-Myc in the developing adult epithelial stem cells and c-Myc in turn promotes the adult stem cell formation and/or proliferation.

Surprisingly, during intestinal metamorphosis, Mad1 is expressed in the degenerating larval epithelial cells during the early phase of intestinal remodeling and absent in the differentiating adult epithelial cells near the end of metamorphosis when the adult epithelium is formed. Thus, unlike in cell cultures or adult tissues, Mad1 appears to function as a pro-apoptotic gene during normal development, at least in the intestine. Consistently, Mad1 knockout tadpoles have reduced apoptotic cells during T3-induced metamorphosis. It is unclear how Mad1 affects T3-induced apoptosis. Mad1 may directly induce larval epithelial cell death or cause cell cycle cessation, thus inhibiting proliferation and indirectly facilitating cell death when T3 is present.

It is worth pointing out that Mad1 knockout animals develop apparently normally through natural metamorphosis without measurable defects. This is likely because normal metamorphosis take longer time, which allows endogenous compensatory mechanisms to take place, thus ensuring proper development. Similarly, Mad1 knockout has little effect on mouse development.^43^ There are several other Mad genes present in vertebrates, which may compensate for the loss of Mad1 in *Xenopus* or mouse.^1, 2, 6, 13, 14, 43^ It would be interesting to investigate if these other Mad genes participate in frog metamorphosis.

Aside from the effect on T3-induced larval epithelial cell death, removing Mad1 also leads to increased adult intestinal epithelial stem cell proliferation during T3-induced metamorphosis. This appears to be surprising given the lack of Mad1 expression in the proliferating adult epithelial stem cells. On the other hand, the adult epithelial stem cells are formed *de novo* through the dedifferentiation of some larval epithelial cells, although the exact mechanism remains to be determined.^22, 29^ As the removal of Mad1 from the larval epithelial cells reduce T3-induced larval apoptosis, it is temping to speculate that this reduction in larval cell death allows more epithelial cells to gain the opportunity to undergo T3-induced dedifferentiation to become adult stem cells, thus leading to increased adult epithelial cell proliferation. Alternatively, low levels of Mad1 may be normally present in the proliferating adult stem cells and function to reduce cell proliferation. In knockout animals, these low levels of Mad1 are eliminated, thus, leading to increased cell proliferation. In any case, our findings suggest that the balance of Mad and Myc expression in the intestinal epithelium help to control epithelial cell fate determination in the presence of T3.

## Materials and methods

### Experimental animals

Wild-type *X. laevis* or *X. tropicalis* adults were purchased from Nasco (Fort Atkinson, WI, USA) or Xenopus 1 (Dexter, MI, USA). The developmental stages were based on Nieuwkoop and Faber.^44^ Premetamorphic *X. laevis* and *X. tropicalis* tadpoles at stage 54 were treated with 10 nM T3 at 18 °C and 5 nM or 10 nM T3 at 25 °C, respectively. At least three tadpoles were analyzed for each stage or day of T3 treatment. All experiments involving animals were carried out as approved by the National Institute of Child Health and Human Development Animal Use and Care Committee.

### Cloning of Mxd1 (Mad1)

An EST sequence (Gene Bank CV079029.1), found to be highly expressed in the epithelium of *X. laevis* intestine as new stem cells are forming,^28^ was identified to be derived from the *X. laevis* Mxd1 by using multiple rounds of RACE cloning. Briefly, *X. laevis* stage 62 whole intestinal RNA was purified for RACE cloning by utilizing RNeasy Protect Mini Kit (Qiagen, Valencia, CA, USA) and treated with RNase free DNase I (Invitrogen, Grand Island, NY, USA). The first round RACE ready cDNA was generated (Gene Racer RACE Ready cDNA Kit, Invitrogen) by using a gene-specific reverse primer (5′-TGTGTGTTTGAGTGTTGGGAGAATGTAG-3′). Then PCR was carried out by using Prime STAR DNA polymerase (TakaRa, Shiga, Japan) with the forward primer provided by the kit and reverse primers specific to the EST (5′-ACATAGGCCTCAGAAACACAACAATA-3′), followed by PCR with the nested primer (5′-CAAGTCACCGAGGAGTTTCATGACC-3′), resulted in a PCR product of over 1 kb in size. That product was cloned by using TOPO Blunt Cloning Kit (Invitrogen). The plasmid was purified by using a spin Mini-Prep kit (Qiagen), and sequenced. Nested 5′ RACE reactions were subsequently performed by using the same RACE ready cDNA with reverse primers designed from the newly sequenced fragment (5′-ACCTTGCAGGAACGGTCTGTCCCT-3′ and 5′-CACTGTTGTTACTGATTTTAGGCAGCG-3′). The resultant product was again cloned and sequenced and new reverse primers (5′-TTTCACTGGACAAAACCCCTCTCAG-3′ and 5′-TAGTGAGACAAAAGGGCGATGACGT-3′) designed to perform a final RACE reaction on the original RACE ready cDNA. Finally, a nearly 3 kb product was cloned, sequenced and identified as the gene Mxd1, which produces the protein Mad1.

### Expression analysis

Total RNA was isolated from the tadpole tissues using the SV Total RNA Isolation System kit (Promega, Madison, WI, USA). The reverse transcription (RT) reaction was carried out by using the High Capacity cDNA Reverse Transcription kits (Applied Biosystems, Foster City, CA, USA). The qRT-PCR was done as described before.^45^ Briefly, 1 *μ*g total RNA were reverse-transcribed into cDNA using the RT^2^ Easy First Strand Kit (Qiagen). The resulting cDNA was diluted 1 : 10 and the diluted products (2 *μ*l) were subjected to PCR by using a SYBR Green PCR Master Mix (Applied Biosystems) in a 20 *μ*l of reaction solution and the StepOnePlus real-time PCR system (Applied Biosystems) according to the manufacturer’s protocol. The primers used were forward 5′-GCTTCTGGAGGCGGCGGAGTATCT-3′ and reverse 5′-TCTTCAAGCCGTCCCTCTCCTTGCT-3′ for *X. laevis* Mad1, forward 5′-TGGGGTGGAAAGGACGCGCA-3′ and reverse 5′- ACTGCTACTGCTGCTCCAATCCA-3′ for *X. tropicalis* Mad1, forward 5′-CCCACTGAACGACAGCATTTCCAA-3′ and reverse 5′-CGTCAATCTCCTCTTCCTCGTCGC-3′ for *X. laevis* c-Myc, forward 5′-AGCCTCTCCCCTCCTTCCCATCA-3′ and reverse 5′- TTGTGATGGGCTCGTTTGTCTGCC -3′ for *X. laevis* Max, forward 5′-AAGAAGGATCTGGCAGCGG-3′ and reverse 5′-TTTAATGACACCAGTTTCCACA-3′ for *X. laevis* elongation factor-1*α* (EF1*α*), forward 5′-AAGAGGGATCTGGCAGCGG-3′ and reverse 5′- AAGGACACCAGTCTCCACAC-3′ for *X. tropicalis* EF1*α*. The level of Mad1 mRNA was normalized against the level of EF1*α* mRNA for each sample.

### Immunohistochemistry and *in situ* hybridization

A 1024-bp fragment, which includes the full-length coding region and a portion of the 3′-UTR of Mad1, was amplified with Prime STAR DNA polymerase (TaKaRa) and the primer pair 5′-ATGGCGGCCCCGGTCTGTGT-3′ and 5′-CAGTAGGTTACTCTTGCTCCCAGTTGGAG-3′, and inserted into pCR-Blunt II-TOPO cloning vector (Invitrogen). Digoxin-labeled antisense Mad1 probe was generated by transcribing the linearized Mad1 plasmid with the SP6/T7 DIG RNA Labeling Kit (Roche Applied Sciences, Indianapolis, IN, USA). The intestine was isolated from tadpoles at different stages, flushed to remove food residues and fixed in 4% MEMFA for 2 h at room temperature. After removing the fixation buffer, the fixed intestine was incubated in 10% sucrose, 20% sucrose, 30% sucrose and OCT, respectively, for 10 min each, then cut into appropriate length and imbedded in OCT for frozen sections. The intestine was then cut to 10 *μ*m sections with a cryotome, and dried on slide for 2 h at 42 °C.

*In situ* hybridization was carried out according to previous description.^46^ For double staining, slides were fixed in 4% MEMFA for 20 min at room temperature after stopping the *in situ* hybridization staining. The slides were then washed in TE buffer (10 mM Tris-HCl, 1mMEDTA, pH 8.0) for 5 min twice and treated with 10 *μ*g/ml proteinase K (Roche) in 50 mM Tris-HCl, 5 mM EDTA pH 8.0 for 5 min at 37 °C. After washing for 5 min in 1 × TBST (Tris-buffered saline add 0.1% Tween-20) three times, the slides were incubated with blocking buffer (10% normal goat serum in 1 × TBST) at room temperature for 1 h. Anti-PCNA (Novocastra, Newcastle, UK) antibody (dilution ratio 1:100) was added and the slides were incubated at 4 °C overnight. They were then washed for 10 min in 1 × TBST three times and incubated with FITC-labeled secondary antibody (dilution ratio 1:100) (Millipore, Bedford, MA, USA) for 1 h at room temperature. The slides were next mounted with DAPI-containing mount medium (Vector Laboratories, Burlingame, CA, USA). Bright view and fluorescent photographs were taken by using a digital CCD color camera (Retiga EXi Fast 1394, Qimaging, Burnaby, British Columbia, Canada) attached to an optical microscope (BX60, Olympus, Tokyo, Japan).

### Construction of TALENs

TALENs targeting *X. tropicalis* Mad1 were assembled as described.^47, 48^ Briefly, TALENs were designed using TAL Effector-Nucleotide Targeter 2.0 (https://tale-nt.cac.cornell.edu) and assemblies were performed as previously described.^49^ The target sequences of Mad1-TALEN-ELD and -KKR were 5′-TGGCGGCCCCGGTCTGTGTC-3′ and 5′-TAGATACTCCGCCGCCTCCA-3′, respectively.

### TALEN mRNA microinjection

TALEN mRNAs were transcribed *in vitro* using the mMESSAGE mMACHINE SP6 Transcription Kit (Thermo Fisher Scientific, Waltham, MA, USA). In all, 400 pg TALEN mRNAs were injected into *X. tropicalis* embryos at the one-cell stage along with 200 pg of DsRed mRNA (Clontech, Palo Alto, CA, USA). The fluorescent product of the latter was used to identify successfully injected embryos and confirm that the injected mRNA had been translated.

### Mutation analysis and generation of Mad1 knockout tadpoles

Genomic DNA was extracted from whole embryos at stage 35/36 by using DNeasy blood and tissue kit (Qiagen) to check whether tadpoles have mutations in the TALEN-targeted region in Mad1. DNA fragments containing the target site were amplified using the PrimeSTAR Max DNA Polymerase (TaKaRa) and the primers Mad1-F1 (5′-AGAACATTTGGGCGCAAAGAGG -3′) and Mad1-R1 (5′-TGTGGGATGCTGGGAGTTGTAG -3′) for 25 cycles (98 °C, 10 s; 60 °C, 5 s; 72 °C, 5 s). The second round of PCR was performed by using the PCR product and Mad1-F2 (5′-CACCCCCTGTCACACAGTACAA-3′) and Mad1-R2 (5′-TGCAGAATACATGGTGAGTGCAG-3′) for 30 cycles (98 °C, 10 s; 60 °C, 5 s; 72 °C, 5 s). The amplified fragments were inserted into the TOPO vector (Invitrogen) and the nucleotide sequences were subsequently determined. To obtain knockout animals, the F0 TALEN mRNA injected animals were raised to maturity and crossed with wild-type animals. The resulting F1 generation tadpoles were genotyped by tail clipping to identify the tadpoles carrying mutations. Tadpoles carrying frame-shift mutations were raised to adulthood for mating. A F0 male containing mosaic frame-shift mutations or a F1 male with a 10-nucleotide deletion in the Mad1 male were mated with a F1 female with 10-nucleotide deletion to obtain F2 offspring with mutated Mad1.

### EdU labeling

EdU staining was performed as described.^50^ Briefly, 1.25 *μ*l of 10 mg/ml EdU were injected into stage 54 tadpoles with or without T3 treatment. Thirty minutes after injection, the tadpoles were killed, and the intestine was fixed in 4% MEMFA and processed for paraffin sectioning. Tissue sections cut at 7 *μ*m were subjected to EdU staining by using the Click-iT Plus EdU Alexa Fluor 594 Imaging kit (Thermo Fisher Scientific) according to supplier’s instructions. EdU-positive areas in epithelium were measured by using ImageJ software (National Institutes of Health, Bethesda, MD, USA).

### MGPY staining

Sections were stained with MGPY (Muto, Tokyo, Japan), a mixture of methyl green, which binds strongly to DNA, and pyronin Y, which binds strongly to RNA, for 5 min at room temperature. Adult epithelial stem/progenitor cells were intensely stained red because of their RNA-rich cytoplasm.^21, 51^

### TUNEL assays

TUNEL assays were performed by using DeadEnd Colorimetric TUNEL System (Promega) as described.^52^

### Statistical analysis

Data are presented as mean±S.E. The significance of differences between groups was evaluated by one-way ANOVA followed by Tukey’s multiple comparison test or Student’s *t*-test using Prism 5 (Graph Pad Software Inc., San Diego, CA, USA).
